# Hypervascular Lesion in a Cirrhotic Liver: A Case Report

**DOI:** 10.14740/gr630w

**Published:** 2014-12-27

**Authors:** Lucas Carvalho Santos, Juliana Bianchi, Suzzanna Ingryd Goncalves Souza, Luiz Arnaldo Szutan

**Affiliations:** a Faculdade de Ciencias Medicas da Santa Casa de Sao Paulo, Sao Paulo, Brazil; b Surgery Department, Faculdade de Ciencias Medicas da Santa Casa de Sao Paulo, Liver and Portal Hypertension Group of Irmandade da Santa Casa de Misericordia de Sao Paulo, Sao Paulo, Brazil

**Keywords:** Liver, Hypervascular lesion, Hepatocellular carcinoma, Focal nodular hyperplasia

## Abstract

Hepatocellular carcinoma (HCC) is the leading cause of death among patients with cirrhosis. Therefore, a focal hepatic lesion in a patient with cirrhosis must always be investigated for its high risk of cancer. However, when hepatic lesions in an imaging exam do not present the typical characteristics of a malignant or a benignant tumor, diagnosis may be a challenge. The biopsy can be used in these circumstances, but, as shown by this case, even that can be misleading. A 54-year-old male patient with cirrhosis presented with abdominal pain, jaundice, nausea and vomits. He performed a biopsy at another service, with the result being focal nodular hyperplasia (FNH). He presented adequate hepatic function, and alpha-fetoprotein level was 6.4. Upon first consultation, we required the slides to be brought to our service and reviewed. Our revision also showed no signs of malignancy. Magnetic resonance imaging showed a large hepatic tumor in segments V and VI, predominantly exofitic, with a central scar. The tumor was surgically removed, and its dimensions were 14 × 10 × 9 cm. Microscopic examination revealed an HCC. Even though histological diagnosis was not necessary to indicate surgery, due to its exofitic nature and adequate hepatic function, we discuss the diagnostic characteristics of both HCC and FNH that could help other medical groups in cases where the position of a liver tumor could make the decision to operate more difficult.

## Introduction

Hepatocellular carcinoma (HCC) is a common malignant tumor of the liver, but the differential diagnosis with benign tumors of the liver may be a challenge. When typical findings of these tumors are not present in imaging exams, it may be required to extirpate the tumor without previously knowing the diagnosis, or resort to a biopsy. However, as shown by this case report, even the biopsy may be misleading.

In this case report, we present the case of a patient with alcoholic cirrhosis that developed abdominal pain. Imaging exams showed a large mass originating from segments V and VI of the liver. A biopsy was performed and showed signs suggestive of focal nodular hyperplasia (FNH). However, after the tumor was surgically removed, it was identified as HCCs.

## Case Report

A 54-year-old male patient, with previous diagnosis of alcoholic cirrhosis, presented with abdominal pain for 5 years, in the right hypocondrium, associated to jaundice, nausea, vomits and the loss of 12 kg of weight in the last year. The pain had no relation to eating. He reported a previous hospitalization in another hospital to perform a liver biopsy, which showed signs of FNH. He was referred to our service due to the lack of a proper structure to operate this patient. Upon the first consultation, we requested the slides of the biopsy to be brought to our service for a review, which revealed an altered lobular structure due to portal fibrosis, forming large fibrous septa, and no signs of a malignant tumor.

**Figure 1:**
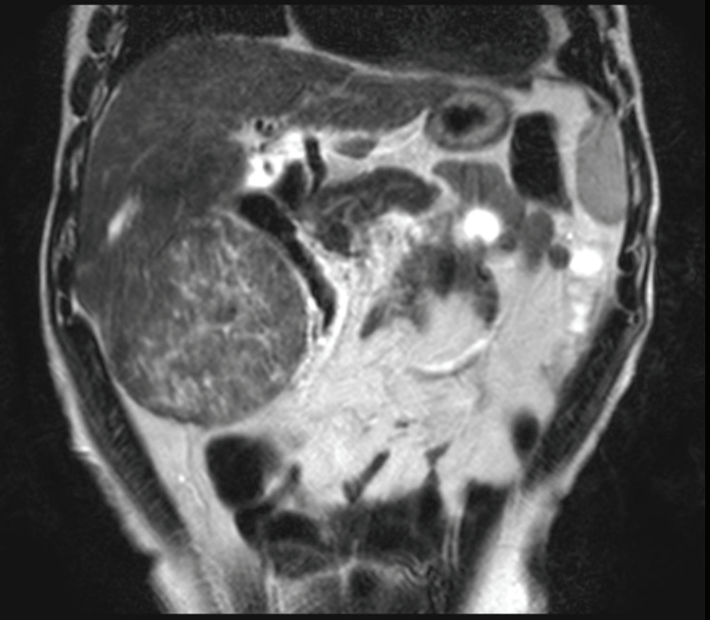
Abdominal contrasted MRI, T2-weighted, showing the tumor. It presented close proximity to the right kidney and the hepatic flexure of the colon.

We performed a magnetic resonance imaging (MRI) exam (Fig. 1), which revealed a normal sized liver, and an expansive lesion originating from segments V and VI, heterogeneous and encapsulated, with well-defined limits, and heterogeneous enhancement by the contrast, similar to normal parenchyma. It also presented areas of central scar and some foci of necrosis.

Biochemical analyses performed at this time showed that his alpha-fetoprotein value was 6.4. With this finding, the biopsy and the imaging exam, his diagnosis was set as FNH.

Other biochemical analyses performed were aspartate transaminase: 34, alanine transaminase: 27, albumin: 2.8, direct bilirubin: 0.5, indirect bilirubin: 0.3, international normalized ratio: 1.51. Given he had no signs of encephalopathy or ascites, he was considered Child-Pugh A. Hepatitis B and C serologies were negative.

**Figure 2:**
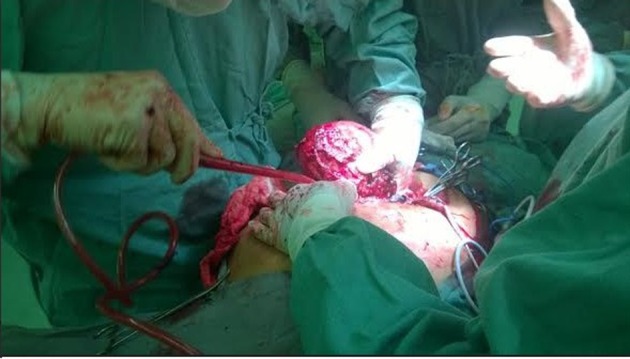
Intraoperative picture of the tumor.

Surgical intervention was performed because of the symptoms. The tumor was successfully extirpated (Fig. 2). After his recovery in an intensive care unit, he was discharged with return appointment for 2 weeks later.

**Figure 3:**
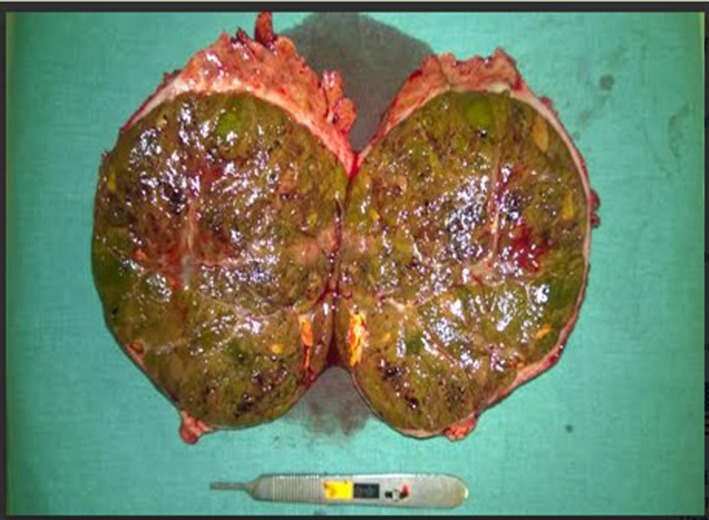
Tumor sectioned in half.

**Figure 4:**
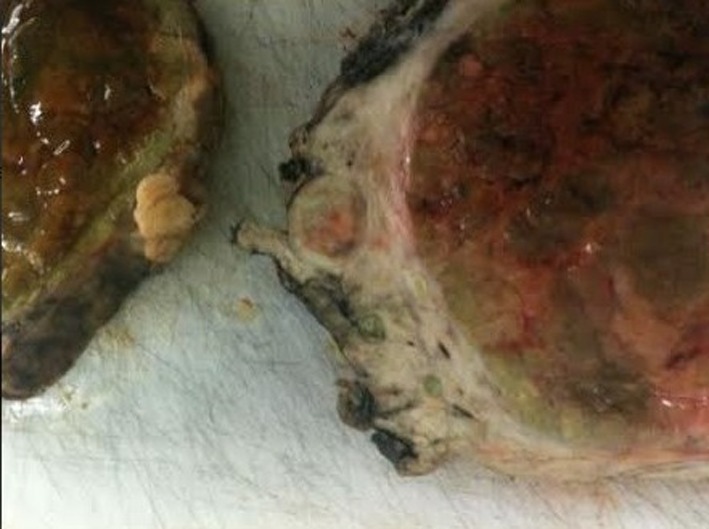
Image of the tumor sectioned in half, with detail to one of its nodules. It is possible to observe a nodule that microscopic analyses could not conclude whether it was completely extirpated from the patient.

**Figure 5:**
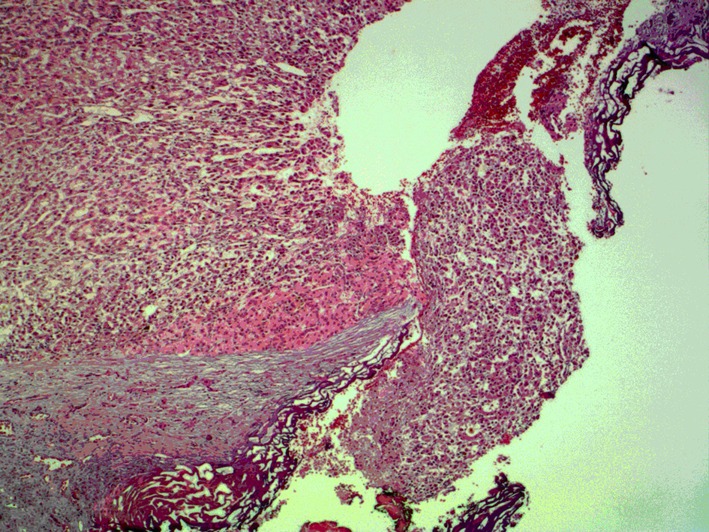
Microscopic view of the part of the tumor where the nodule was sectioned. It is possible to observe that cancerous cells were present in the nodule, and there is no margin of normal cells, which leads us to believe that there are still cancerous cells in the organ.

Macroscopically, the dimensions of the tumor were 14 × 10 × 9 cm, and weighed 701 g. Upon being sectioned in half (Fig. 3), a margin of normal parenchyma of 1.4 cm was observed, with nine satellite nodules inside the margin (Fig. 4), with the same characteristics of the main tumor. Microscopic examination revealed an HCC (Fig. 5), with pseudoglandular areas, moderately differentiated (stage two).

## Discussion

The relationship between cirrhosis and HCC is well established. HCC is the sixth most common cancer worldwide, and is the leading cause of death among patients with cirrhosis [1]. Therefore, upon discovery of a hypervascular lesion in a patient with known cirrhosis, it is important to consider HCC as the first diagnosis, until proven otherwise.

The imaging exam of choice for this patient was the MRI. Several studies have demonstrated the superiority of MRI in both lesion detection and characterization for focal hepatic lesions when compared to CT [2]. FNH tipically presents a central scar, which is hypointense in T1-weighted images and hyperintense in T2-weighted images [3], as was the case with this patient, though it can be hypointense in up to 25% of the cases [4]. The lesion itself is typically iso- or hyperintense in T1-weighted images, being hypointense in only 6% of the cases, while in T2-weighted images it is iso- or slightly hyperintense in 94-100% of the cases [4], which is the pattern presented by the MRI exam in this case. The lesion is also solitary in 75-80% of the cases [3]. FNH does not have a tumor capsule, but a pseudocapsule may be present, resulting from the compression of surrounding liver parenchyma, perilesion vessels and inflammatory reaction [5]. A true tumor capsule, on the other hand, is present in 60-80% of the cases of HCC [5]. This capsule mainly consists of fibrosis and has low signal intensity on both T1- and T2- weighted images [5]. The lesion in this case presented an area around it that could not be distinguished between a true capsule or a pseudocapsule, and was isointense in both T1- and T2-weighted images. It also presented a very heterogeneous enhancement on T2-weighted images, which is uncommon for FNH.

The size of the tumor, 14 × 10 × 9 cm, also contributes to the diagnostic doubt. Even though FNH can present itself as a volumous abdominal mass, it is usually of smaller size. One study found that only two out of 86 patients (2%) had a tumor with a diameter greater than 10 cm [6].

This patient presented himself to us already with a biopsy, which showed no signs of malignancy. The biopsy was brought to our service and reviewed, and the result was the same. The material was obtained through open liver biopsy, and it is possible it only sampled the area around the lesion. Classic FNH is characterized by the presence of abnormal nodular architecture, malformed vessels and cholangiolar proliferation, and nonclassic is characterized by the presence of two of the previous criteria, but always need to present cholangiolar proliferation [5]. Considering the biopsy revealed an altered lobular structure, with cholangiolar proliferation, but no malformed vessels, the lesion could be classified as a nonclassical FNH, which is the one in which the correct pre-operative diagnosis is the most dificult to establish with imaging exams [6]. Furthermore, nonclassical FNH usually presents a heterogeneous macroscopic appearance, as shown in the MR image, but it also usually lacks a central scar [5].

A further refinement in the exams performed in this case that could have contributed to the diagnosis would be the hepatic tissue-specific MR contrast media, such as superparamagnetic iron oxide (SPIO) or gadoxetic acid. Iso- or hyperintensity, or sometimes ring enhancement, of the lesion in the hepatocyte image of Gd-EOB-DTPA-enhanced MR images is a very specific finding of FNH [3].

In the present case, due to the satisfactory hepatic function, and the predominantly exofitic location, surgery was indicated independently of hystological diagnosis, and therefore it did not present doubts on the conduction of the case. But a different case, in which determining malignancy of a tumor in a central location of the liver is a challenge, may benefit from this discussion about the diagnostic characteristics of liver tumors, which could contribute to the decisions made by a different medical group.
